# A Review on Combination of Ab Initio Molecular Dynamics and NMR Parameters Calculations

**DOI:** 10.3390/ijms22094378

**Published:** 2021-04-22

**Authors:** Anna Helena Mazurek, Łukasz Szeleszczuk, Dariusz Maciej Pisklak

**Affiliations:** 1 Department of Physical Chemistry, Chair and Department of Physical Pharmacy and Bioanalysis, Faculty of Pharmacy, Doctoral School, Medical University of Warsaw, Banacha 1 Str., 02-093 Warsaw, Poland; annamazurek21@gmail.com; 2 Department of Physical Chemistry, Chair and Department of Physical Pharmacy and Bioanalysis, Faculty of Pharmacy, Medical University of Warsaw, Banacha 1 Str., 02-093 Warsaw, Poland; dpisklak@wum.edu.pl

**Keywords:** aiMD, ab initio, molecular dynamics, NMR, GIPAW, GIAO

## Abstract

This review focuses on a combination of ab initio molecular dynamics (aiMD) and NMR parameters calculations using quantum mechanical methods. The advantages of such an approach in comparison to the commonly applied computations for the structures optimized at 0 K are presented. This article was designed as a convenient overview of the applied parameters such as the aiMD type, DFT functional, time step, or total simulation time, as well as examples of previously studied systems. From the analysis of the published works describing the applications of such combinations, it was concluded that including fast, small-amplitude motions through aiMD has a noticeable effect on the accuracy of NMR parameters calculations.

## 1. Introduction

“It is difficult to overemphasize the importance of magnetic resonance techniques in chemistry”—with those words begins *Calculation of NMR and EPR Parameters* [1], a book which over time has become an irreplaceable and highly appreciated guide for researchers combining experimental and theoretical aspects of NMR in their everyday work. Since 2004—the year of the first edition—this opening line has become even more real and indisputable. The number of examples of such combinations in which experimental and theoretical NMR complement and complete each other is constantly increasing. The kind and size of the substances being the objects of NMR computational studies are unlimited, from very small organics up to biological macromolecules and polymers. Calculations play an even more important role in the inorganic and organometallic fields, where empirical interpretations are far more difficult.

Though the possibility of combining the NMR experiments and calculations is fascinating, which is particularly visible when looking at the new applications of such a tandem, this is not what this review explores. Our aim was to focus on the particular type of NMR parameters calculation methodology that includes the combination of the ab initio molecular dynamics (aiMD) followed by the quantum mechanical (QM) NMR parameters calculations in which the trajectory generated during the MD is utilized in the NMR calculations. In the aforementioned book, the chapter describing such a combination is the shortest one, presenting only a few examples on very small molecules and expressing the hope of the authors that such a combination will become more affordable and more frequently applied in the future. Now, after almost twenty years, it is justified to state that those hopes have borne fruit, which we try to prove in this review. This improvement was possible mainly due to the fact that in recent years, there has been a rapid increase in the computational power of commonly used units—MD calculations are now commonly performed not only using CPU but also GPU. Besides, the access to software enabling QM calculations on different systems has significantly increased and the implementation of aiMD simulations can be found in many of those programs, which is also discussed in this article.

First, it should be stated what this review does not cover. Surely, we do not describe the theory behind NMR parameters calculations as there are many excellent books and reviews describing those aspects, both the basics and origins, as well as the recent advances. Furthermore, the concepts of aiMD are rather briefly mentioned with a particular attention on the aspects related to NMR parameters calculation for similar reasons, and a focus on those methods that have been already used in combination with NMR computations. Additionally, the studies combining NMR calculations and the classical MD—employing the potentials calculated using molecular mechanics—are described. It is not that there are no such works, on the contrary—the number of those kind of studies is increasing rapidly, which even resulted in the launch of the Special Issue of IJMS titled *Combined NMR Spectroscopy and Molecular Dynamics Studies for Infectious Diseases,* devoted to the studies “describing comprehensive characterization of both structural and dynamical processes in protein complexes and interactions, which is critical for fully understanding the mechanism behind many biological processes” [2].

Instead, we summarize the previously published works on the application of combined aiMD and NMR parameters calculations. We present the reasons why the authors of those publications have decided to perform those rather demanding calculations instead of the “simple” NMR computations for the energetically minimized structures. This review is focused on the practical aspects, such as the description of the software that can be used to perform the MD and NMR calculations and in some cases, both of them. Furthermore, we present the advantages as well as disadvantages of such an approach and compare its accuracy with others used in the field of NMR parameters calculations.

We believe that gathering previously published information can be beneficial for several reasons. First, it enables a convenient overview of the applied parameters such as the DFT potential, MD type, time step, or total simulation time, etc. Then, we try to convince the readers that due to the development of both software and hardware, such an approach may be not difficult to use and feasible even in the studies that are not primarily focused on NMR calculations. As mentioned earlier, due to the huge variety of the studied systems, there is no universal solution or approach to combine the aiMD and NMR calculations. However, the descriptions of successful applications of such combination presented in this work can surely serve as a guide, especially for the readers that are more or less familiar with the NMR parameters calculations.

This review is organized in three sections. In the first, the basics of MD, with a particular stress on aiMD, is summarized. In the second one, the most important and interesting aspects of the reviewed articles that have described the application of combined aiMD and NMR calculations are presented. In the next section, those studies are briefly discussed to provide a general overview, followed by concise conclusions.

## 2. Ab Initio Molecular Dynamics Theory

### 2.1. Molecular Dynamics Simulations

Molecular dynamics (MD) simulation is a well-established technique used for the study of various chemical substances and mixtures in any state of matter and almost at any temperature and pressure conditions [3,4,5,6,7]. MD is sometimes described as “computational molecular microscope”, while in fact, MD is more than just an in silico microscope as it can be used to determine not only structural but also energetic and thermodynamic properties [8], as well as to improve the accuracy of NMR parameters calculations, which is discussed in the next section.

Molecular dynamics methods, regardless of the objects being modeled and the method being used, are, in their basic assumptions, similar. The simulation starts with an initial configuration of the system and energy minimization through the optimization of the positions of all atoms [9]. Subsequently, the forces acting on each atom are then calculated and used in equations of motion to update the configuration. This process is repeated to generate a trajectory—a contiguous set of configurations obtained during the time evolution of a studied system.

One of the most challenging aspects of MD simulations is the calculation of the interatomic forces. In classical molecular mechanics-based simulations, they are computed from empirical potential functions, which have been previously parameterized to reproduce experimental or accurate ab initio data of small model systems [10]. Even though these empirical potentials have been created with great effort to be as accurate as possible, usually the transferability to systems or regions of the phase diagram different from the ones to which they have been fitted is restricted and may lead to significant inaccuracy [11]. Moreover, some of the most important and interesting phenomena of modern physics and chemistry are intrinsically nonclassical but quantum [12]. Therefore, a first-principles-based approach, such as aiMD, where the forces are calculated on-the-fly from accurate electronics structure calculations, is very attractive as many of these limitations can be removed. However, the increased accuracy and predictive power of such simulations comes at a significant computational cost that was, for a long time, not affordable for most researchers. For this reason, density functional theory (DFT) is so far the most commonly employed electronic structure theory in the context of aiMD. However, it is important to note that aiMD is a general concept that can be used in conjunction with any electronic structure method [13].

### 2.2. Ab Initio Molecular Dynamics (aiMD)

In almost every method of molecular modeling, in order to reduce the computational complexity and in consequence the time of calculations and hardware requirements, some approximations must be applied, while maintaining the predictive accuracy. In the field of aiMD, there are two most commonly used approximations. The first one, called the adiabatic approximation, assumes that the electronic wave functions adapt quasi-instantaneously to a variation of the nuclear configuration. The second one, named the Born–Oppenheimer approximation, assumes that the electronic and the nuclear motions are separable due to the significant difference between nuclear and electronic masses [14].

Two of the most commonly used methods of aiMD simulations in the studies combined with NMR parameters calculations are the Born–Oppenheimer (BO) MD [15] simulations—in which the forces at each timestep are calculated quantum mechanically and the classical Newtonian equations of motion are being solved—and the ab initio Car–Parrinello (CP) MD [16] simulations, where quantum mechanical calculations are performed at each step of the simulation to solve the Car–Parrinello equations of motion. However, in the literature, the examples of successful applications of more computationally demanding Path integral MD (PIMD) [17] simulations can be found, as well.

In BOMD, it is assumed that the adiabatic and the Born–Oppenheimer approximations are valid and that the nuclei follow a semi-classical Newton equation whose potential is determined by the Ehrenfest theorem. It is further postulated that the electronic wave function is in its ground state, implying the lowest energy. In Born-Oppenheimer MD (BOMD), the potential energy is minimized at every MD step. Nevertheless, due to the inclusion of the BO approximation, the electronic and nuclear subsystems are fully decoupled from each other. Due to the adiabatic separation, there are no additional restrictions on the maximum permissible integration time step, which hence can be chosen up to the nuclear resonance limit.

**Figure 1:**
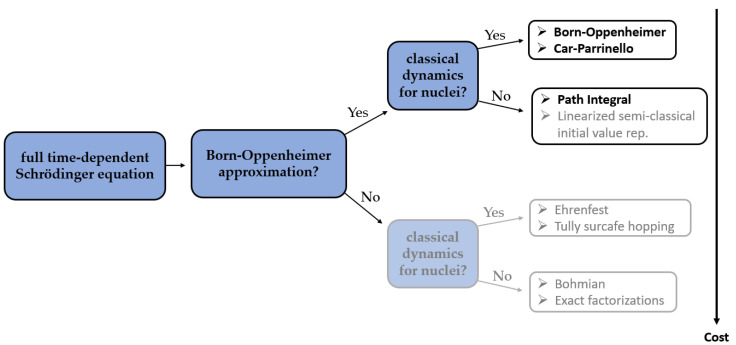
The major differences between the methods of aiMD simulations [18]. Bolded methods have already found their application in combination with NMR parameters calculations.

In CPMD, a coupled electron-ion dynamics is performed, wherein the electronic degrees of freedom are added to the Lagrangian as classical ones. At variance to BOMD, the computational cost to compute the nuclear forces in each aiMD step is now much reduced since no SCF (Self-Consistent Field) cycle is required to ensure that they are consistent with the instantaneous energy, and to force the electrons to adiabatically follow the nuclei. However, in the CPMD, the required time step is usually significantly shorter than in BOMD, which increases the computational time of CPMD simulation. An analysis of previously reported studies shows that it is very hard to unambiguously determine if either BOMD or CPMD is to favor. The differences between the results obtained using those two methods are often rather subtle and depend largely on the particular application (Figure 1).

The ab initio path integral technique is based on the formulation of quantum statistical mechanics in terms of Feynman path integrals. Contrarily to the previous approaches, the path integral molecular dynamics (PIMD) allows for a quantum formulation which includes, in addition to the electronic degrees of freedom, their nuclear counterpart. Such an approach is therefore recognized to be more accurate than BOMD or CPMD, especially for systems containing light nuclei. Unfortunately, conventional PIMD simulations require a considerable computational cost, which is the reason why they are not so popular in combination with NMR parameters calculations yet.

Integrating equations of motion allows the exploration of the constant-energy surface of a system. However, most natural phenomena occur under conditions where the system is exposed to external pressure or exchanges heat with the environment. Under these conditions, the total energy of the system is no longer conserved and extended forms of molecular dynamics are required [19,20].

Depending on which state variables (the energy E, number of particles N, pressure P, temperature T, and volume V) are kept fixed, different statistical ensembles can be generated. Microcanonical (NVE) ensemble in which the energy is conserved is the natural ensemble to simulate molecular dynamics. Canonical (NVT) ensemble requires the system to be in contact with a heat bath so that the states of the system will differ in total energy. Isothermic-Isobaric (NPT) ensemble is particularly interesting because most experiments are usually done at constant temperature and pressure, which enables more accurate modeling of conformational changes. Several methods are available for controlling temperature and pressure by means of thermostat and barostat algorithms [21,22].

### 2.3. Combining the aiMD and NMR Parameters Calculations

NMR spectroscopy is currently one of the basic tools for the structural analysis of various systems and is widely used in the studies of liquid, solid, and gas phases. In the experimental approach, the basic NMR parameter is the chemical shift (δ) characterizing the position of the resonance line in the NMR spectrum. From the physical description point of view, it is directly related to the shielding parameter (σ), described by a second order tensor. The position of the line in the NMR spectrum can be directly related to the isotropic value as determined by the trace of the tensor, but the individual components influence the shape of the lines. While in solution, due to the short correlation times, the screening parameters are averaged to the isotropic value; in the case of solid-state NMR measurements, the full tensor can be usually determined, which allows a deeper analysis of the molecular structure and interactions. The QM calculations methods allow to determine the full tensor and analyze not only the isotropic parameter, but also the individual components of the anisotropy tensor.

Another NMR parameter that can be obtained from NMR spectrum is the indirect spin-spin J coupling, which is highly dependent from molecular geometry and widely used in conformational analysis. For quadrupole nuclei (with spin I > ½), the additional quadrupole coupling has a strong influence on the position and shape of the signal line in the experimental NMR spectrum. All these parameters allow for a deep insight into the structure of molecular systems. The use of QM computational methods allows for the calculation of these parameters, which not only simplifies the analysis of NMR spectra, but also allows to translate the experimental parameters into structural aspects of the analyzed molecular system.

NMR shielding tensors may be computed using multiple methods, such as the Continuous Set of Gauge Transformations (CSGT) [23], Gauge-Independent Atomic Orbital (GIAO) [24], Individual Gauges for Atoms In Molecules (IGAIM, a slight variation on the CSGT method) [25], Single Origin (SO), or Gauge Including Projector Augmented Wave (GIPAW) [26]. The similarities and differences of these approaches are not discussed in this review so as not to stray from the main topic. Such quantum chemical calculations are typically performed using static, optimized structures at 0 K, and thus neglecting zero-point motion and not accounting for the thermal dynamics, which can lead to significant discrepancies between computed and experimental data. This is particularly visible in solutions, where the configurations of neighboring molecules change dynamically, resulting in the dynamic changes of the intermolecular contributions to shielding. Therefore, dynamic averaging should be taken into account, even when there are no significant strong intermolecular interactions.

In order to somehow correct the computational results to include the effects of dynamics, various scaling methods have been proposed. For example, chemical shift anisotropies have often been found to be overestimated by DFT calculations, and several scaling factors ranging from 0.76 to 0.95 [27,28] and depending on the studied structures have been proposed. Such an approach may be quick and easy to implement, but if the discrepancies between calculated and experimental data result at least partially from the neglect of the dynamics in the calculations, then applying such scaling factors removes potential information about dynamics. Therefore, understanding how vibrational motions affect NMR tensor parameters may lead not only to a better agreement with experiment, but also to a proper description of the dynamics in the studied systems. It is not surprising, then, that the number of successful and non-routine studies combining aiMD and NMR parameters calculations is increasing constantly; to prove this statement, some of them are discussed in more detail in the next section.

## 3. Summary of the Chosen Studies Applying Both aiMD and NMR Parameters Calculation

AiMD and DFT-based NMR calculations are performed in solution, solid, and in glass states of matter. The diversity of systems subjected to this computational method along with applied software and methods details are gathered in Table 1. All the studies from the Table 1 are briefly discussed below.

### 3.1. Studies in Liquid State

Solution is the most common environment of MD calculations described in this review, as most NMR experiments are conducted in the liquid phase. However, the fact of doing calculations in a liquid state introduces an important question: how to treat a solvent.

To solve this question, two approaches are available: implicit (continuum) and explicit solvation models. According to the former, a solvent is perceived as a homogenously polarizable medium. This model is usually applied in both the molecular mechanics and ab initio molecular dynamics simulations [57]. Two most often used implicit solvent models are: PCM (Polarizable Continuum Model) [58] and COSMO (COnductor-like Screening Model) [59]. These methods are computationally not very demanding, but unfortunately, they do not allow simulating the local fluctuations around a solute.

To do so, the explicit model must be applied. It is significantly more computationally demanding, requiring fitting methods and parametrization if polarizable force fields such as the AMOEBA are used [60]. The explicit solvation model finds application also in quantum mechanical calculations. It is often used for ion-solvent clusters, which are an area extracted out of a bigger system that cannot be computed with an explicit model [61].

#### 3.1.1. Analysis of Pure Solvents

Already a decade ago, a close investigation was performed on the most common solvent, namely: water. The study concentrated on defining which calculational factors (basis set, functional, pseudopotential) influence the aiMD-NMR approach the most and at which stage (aiMD simulation or calculation of the aiMD-based NMR parameters) is this effect the strongest. The results show that the choice of a DFT functional has a critical impact on the generated data. However, this effect is observed almost only in the process of the aiMD simulation and therefore in the process of snapshots’ generation. On the contrary, for calculation of the NMR parameters, the influence of a type of functional is almost negligible. This relatively very early work from 2010 was concentrated on purely technical aspects of combining the aiMD and DFT-NMR calculations. During the past 10 years, the computational possibilities have increased and therefore, the variety of research objects has strongly expanded.

The example which plainly shows the high accuracy of aiMD calculations, even if applied in a complex system, is a recent work [62]. In this study, on the basis of data derived from CPMD, the H-H bond distances between ethylene glycol and water within their mixture without any other additives were calculated. Different conformations of the donor-acceptor pairs were generated and the CPMD trajectories satisfying the experimental NMR data were collected. This study is a good example that aiMD has a practical use when a complex structure of the hydrogen-bonded liquid must be analyzed. Moreover, via comparison with the experimental NMR, this work underlines the accuracy of the aiMD calculations.

#### 3.1.2. Implicit Solvent

For the aiMD performed in a liquid environment, the implicit solvent model is the most widely used one.

The one group of reasons for applying the “implicit solvent—aiMD—DFT-NMR” model is the need to perform the proper spectral assignment. While for this reason QM NMR calculations on static structures (without aiMD) can be used as well, it was found that the inclusion of aiMD can increase the accuracy and thus reliability of such an assignment. Furthermore, with the aid from aiMD it is also possible to confirm or even propose the mechanism of the dynamical process observed experimentally using liquid-state NMR. It should be mentioned that these three reasons are arranged in a timeline from the oldest to the newest. On the basis of this literature research, it is plainly visible how, over time, the computational possibilities evolved along with the raise in the computational power. Below, some examples of such implicit solvent calculations are presented.

Liquid nitromethane is the commonly applied reference compound in the ^15^N NMR experimental analysis. In order to convert the calculated chemical shielding into experimentally observed chemical shifts for a studied compound, the information on those parameters for a reference compound is essential. For a long time, in order to do such a conversion, a gas state nitromethane was modeled, which caused some inaccuracies due to the inability to model the intermolecular interactions. In the study of Gerber and Joliboi [41], thanks to compilation of computational methods including the aiMD and the implicit solvent model, the accurate theoretical^15^N spectrum of liquid nitromethane was obtained.

Protons exchange during diffusion of phosphoric acid in hydrogen-bond clusters could not be fully analyzed in an experimental way [51]. That is why a theoretical study based on the reliable calculation protocol was used in order to propose the underlying mechanism. Accepted by the authors as equivalently accurate as the experiment, the vibrational and ^1^H NMR spectra based on the BOMD trajectories were simulated. The results confirmed the correctness of the proposed theoretical mechanism. It suggests that for protonated H_3_PO_4_ clusters, structural diffusion can proceed without the reorientation of H_3_PO_4_ molecules.

The study that analyzes the influence of intramolecular charge transfer and Nuclear Quantum Effects on intramolecular hydrogen bonds (IHB) in azopyrimidines can serve as another example [29]. Two IHB models were proposed. Their stability can be compared thanks to the observation of a hydrogen atom dynamics in the aiMD simulation. Only the combination of the experimental and aiMD data enabled the authors to get access to the detailed information on the variations of hydrogen bond geometries upon hydrogen-to-deuterium isotope exchange.

The thorough computational analysis of the relativistic, solvent, and dynamic effects on the ^1^H NMR chemical shifts of iridium polyhydride complexes revealed that in the experiment, two NMR hydride signals were inversely assigned [39]. The reason was probably the high complexity of the analyzed [Ir_6_(IMe)_8_(CO)_2_H_14_]^2+^ system. The authors concluded their work with a statement that with aiMD at hand, there exists a reliable tool to check the correctness of a traditional NMR signals assignment.

The detailed analysis of ^23^Na shielding constants in the methylamine solution of [Na^+^ [2.2.2]cryptand Na^−^] [38], in combination with other experiments, raises up the topic of the signals’ shape in the NMR spectra, electric field gradient values (see: Section 3.1.4) and ion’s core valence shell. In this case, such an approach delivers, as the authors stated, “a complete picture of the NMR of Na^-^ in the cryptand—methylamine system”.

The next example is the analysis of the solvent solvation of Li and Li dissociation from sulfide chains in polysulfide clusters in a nonaqueous mixture of 1,3-dioxolane and 1,2-dimethoxyethane [49]. A couple of details concerning the lithium exchange dynamics between the solvent and the polysulfide species have been explained. According to the article, this data is essential for the design of electrolytes, and it could have not been gathered if aiMD and DFT-NMR had not been used. This study is particularly interesting because in the aiMD, not only temperature but also pressure factor was included and simulations were performed at the NPT ensemble that simulates the real experimental conditions.

#### 3.1.3. Explicit vs. Implicit Solvent Model

Approximately a decade ago, when the computational power reached a certain level allowing more common application of explicit solvent calculations, the research comparing the implicit and explicit solvation models has become popular. A couple of such examples concerning aiMD and NMR parameters simulation are available, as well. Although already a decade has passed, the conclusions resulting from this research are still valid. In all of the works cited below, the cluster method for the explicit solvent model was applied.

Basic comparative studies were performed in [43,44]. The former compares the explicit solvent model in aiMD and implicit solvent model in classical MD. The authors put it straight that the application of aiMD with explicit solvent and quantum-based NMR calculations ended in almost perfect agreement with experiment.

A more comprehensive study is presented in [44]. Here, the ^7^Li NMR chemical shielding of hydrated Li^+^ was calculated in very different environments: in a gas state, with a static method in liquid with implicit solvation model (PCM), and with a quantum-based dynamic method (BOMD) in liquid with explicit solvation model performed on big clusters. In the article, it is plainly stated that the implicit PCM solvent model is insufficient to correctly simulate Li shielding. Out of the three analyzed approaches, only the aiMD-cluster explicit one combined with the DFT-NMR calculations delivered spectra that were well-comparable with the experiment.

The above-described example refers to a simplest model possible: water solution with a small ion. Much more complications can be potentially caused by heavy atoms.

A good example are platinum derivatives. In one of the papers, the J-coupling constants of cisplatin derivatives were investigated with the aiMD combined with DFT-NMR calculations [46]. Both the explicit and implicit solvation models were used. The conclusions were drawn that a good agreement with the experimental data is obtained only when an explicit solvent, a hybrid functionals, and a dynamic approach (aiMD) are applied simultaneously. Results of similar accuracy could be obtained with COSMO (implicit solvent model) only if the platinum solvent-accessible radius was chosen carefully, which was very time consuming. What is more, this study supports the existence of a partial hydrogen-bond-like inverse-hydration-type interaction resulting in a weak ^1^J(Pt···H_water_) coupling between the complexes and the coordinating water molecule.

Another study that concentrated on the mercury derivatives was constructed in the same way. It described simulations in gas phase, in COSMO solvent model, and in explicitly solvated BOMD simulation [47]. This study ended with similar conclusions, as well. It also pointed out that even better results could be obtained if a hybrid functional was applied for both aiMD and J-coupling simulations. At the time when the article was published, such an approach was too computationally demanding for the authors to undertake.

#### 3.1.4. Calculation of the Quadrupolar NMR Spin Relaxation Rates and Coupling Constant from QM Calculations of an Electric Field Gradient (EFG) with Explicit Solvent

Another NMR parameters that can be calculated using QC methods preceded by MD simulations are electric field gradient (EFG) tensors. The EFG tensors obtained that way can be then used to calculate the quadrupolar relaxation rates or quadrupolar coupling constants.

**Figure 2:**
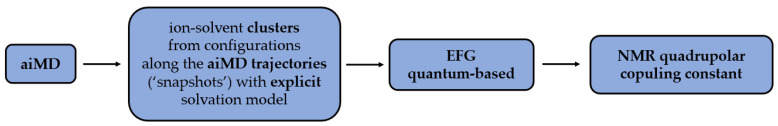
One of approaches used to calculate EFG.

Firstly, the data are obtained from the aiMD trajectories via ion-solvent clusters [63]. The presence of clusters in the process enables usage of the explicit solvation model, which is non-applicable for larger systems. The obtained EFG tensors are then used to calculate the quadrupolar coupling constants (Figure 2). The cluster method addresses the problem of a whole system computation which, in the aspect of quantum calculations, would be computationally too costly.

EFGs calculations could be also performed on the basis of the classical MD, including the explicit solvation model but without making clusters. However, a complicated force field parametrization must be then performed and often some approximations are needed.

Discussed in the paper by [42] is a very transparent example of when the aiMD-cluster and explicit solvent method combined with the EFG calculation used for calculation of the NMR quadrupolar relaxation rates is applied. It concerns the selected ions (^7^Li^+^, ^23^Na^+^, ^35^Cl^−^, ^81^Br^−^, ^127^I^−^) in an aqueous solution.

Another fully quantum mechanics-based study relates to the aqueous solutions of ^127^I^−^, ^131^Xe, and ^133^Cs^+^ [40]. High accuracy of aiMD ensured the correctness of further calculations in this study, which were based on the extracted ion-solvent clusters. The key question of this particular work was which factor contributes more to the EFG tensor value: the solvent or the polarization of a solute.

A comparison between the two above-described methods of EFG calculations, namely those starting from the classical force field-based MD (FFMD) and those starting from the quantum-based MD, as well as the static calculations, was performed on the example of two triazoles computed in different solvents [50]. Both aiMD and classical MD were used with an explicit solvation. This study gave rise to another aspect of the NMR spectra simulation, the line width. In solutions, a line width is proportional to the NMR relaxation rate what cannot be omitted if a quadrupolar nuclei is considered. The quadrupole moment interacts with the EFG and therefore influences the line shape. It has already been shown that in such cases, a static approach is insufficient to properly simulate the line widths [64]. That explains the need to apply the MD. The study of [30] discusses the pros and cons between the usage of aiMD and classical MD in terms of obtainable quadrupolar coupling constants and line widths.

### 3.2. Studies of Glassy Systems

Objects positioned between solutions and solid state described by the aiMD are glassy structures. In these cases, mostly high temperatures are used in the simulations because the investigations concern the conditions around the melting point of the analyzed object. As the inner construction of glass is closer to a crystal solid state system than to a liquid one, the GIPAW method of NMR properties calculations is commonly used for the glass simulations.

As the literature review shows, the structure of a glass in itself is always firstly generated through the classical MD simulation or via application of the Polarizable Ion Model, similarly to a solution. However, afterwards, the obtained system undergoes the BOMD calculation. Usage of both classical and quantum MD in that case is A perfect example that the MD studies do not necessarily have to be limited to only one type of such simulations, force field based or ab initio, but can effectively take advantage of both of them.

For example, for the 0.3 Li_2_O−0.7 B_2_O_3_ glass system, it has been proven that the complex network of this alkali-borate glass cannot be sufficiently described solely by the GIPAW-derived NMR parameters calculated on static, geometrically-optimized structure [52]. This particular system consists of various structural units which, in terms of the computational approach, can be identified precisely enough only when the aiMD is implemented. In this case, due to the rigidness of the system, 764 snapshots must have been derived from the BOMD simulations. Each of them were transformed into a separate structural model for which the GIPAW calculations of the NMR properties were done subsequently.

Another example that confirms the applicability of the BOMD in interpretation of the experimental NMR spectra of glass is the experiment performed on the NaF-ScF_3_system [31]. In this case, not only the network polymerization and formation in the melted glass are defined but also the electrical conductivity in the molten state are computed. The latter is based on the statistical calculations from the ionic BOMD trajectories. One of the aims of the cited work was to prove that a system that was generated via aiMD and validated through the agreement of quantum-based calculated NMR properties with the corresponding experimental data can be successfully used for a reliable calculation of the physicochemical properties, such as density and electrical conductivity. This approach is especially helpful when those data cannot be easily measured experimentally.

### 3.3. Studies in Solid State

#### 3.3.1. Early Works

The most computationally demanding calculations refer to the solid-state systems. Over the last decade, huge improvements occurred in terms of the applicability of the quantum-based MD methods for solids. Already in 2008 Dumez and Pickard, used this method to generate the post-aiMD NMR spectra of small structures such as alanine and aspartyl-alanine [33]. They showed the influence of the chosen DFT functional on the obtained calculations results. They also pointed out that a limiting factor for further research applying aiMD is the computational power.

A couple years later, the computational capabilities have changed a lot. However, taking into account the fact that the calculation cost of a simulation rises quickly with the increase of a structure’s complexity, in a solid state still mostly small systems are investigated. As a consequence, a fair part of the objects undergoing such analysis are amino acids.

Just as in the case of a glass, the aiMD-based NMR is helpful to analyze the molecular motions of the investigated system. Their neglection could lead to very inaccurate calculations of the chemical shifts. Especially, the lowest-frequency vibrational motions bear a large contribution to the temperature dependence of the chemical shifts. On the example of glycine [34], it has been shown that the BOMD approach is sufficient to reproduce the experimental values.

In order to underline the importance of the vibrational motion inclusion, the already quite an old paper by Martin Dračınsky and Paul Hodgkinson should be brought up here [32]. After comparison with the experimental data, it was proven that for the most common nuclei such as ^1^H, ^13^C, ^14^N, ^17^O, and ^35^Cl, aiMD significantly improves the accuracy of the post-simulation calculated quadrupolar couplings. This conclusion is even more appealing if one takes into account the diversity of the structures used in this particular study. Among the chosen examples there were both the simplest amino acids, glycine and alanine, as well as other small organics with aromatic or pyrimidine ring (nucleic acid basis). Such wide variety of the studied solid-state compounds enable the authors to draw some general conclusions on the differences between the NMR parameters obtained using static and aiMD-derived structures, presented in Table 2.

These results concerning the quadrupolar couplings are consistent with the ones obtained in another study from 2017 [65]. There, the discrepancies in DFT calculations of chlorine nuclear quadrupolar coupling constant for ionic chlorides in crystalline systems were thoroughly investigated. It was stated that the quadrupolar couplings calculated from the aiMD snapshots were smaller than those obtained via other computational methods. However, the research proved that in this case, the investigated value depends mostly on the type of applied functional. It was also stated that this influence can be effectively suppressed by the application of scaling factors. As a result, the obtained data for the chlorine agrees well with the experiment and can be used to discriminate between different structures determined via X-ray diffraction, such as L-threonine hydrochloride.

#### 3.3.2. More Advanced Works

Over time, more complex studies involving aiMD combined with DFT-based predictions of the NMR parameters in the solid state have been undertaken.

In one of the works, the effect of the isotope exchange was analyzed [37]. In this study, either proton or deuterium in the ^1^H/^2^H-^15^N bond formed within the guanine-cytosine pair analogues was used. Even if this research’s topic is different than in the previously described systems, here also the bond distance distribution probabilities were obtained thanks to the analysis of the PIMD trajectories. Consequently, this enabled the prediction of the nitrogen shieldings. This, in turn, delivered the answer for the question of the study. The drawn conclusion was that the inter-base proton transfer reaction does not take place in the investigated system. Such information was crucial for further design of the guanine-cytosine pair analogues which could potentially serve as medication.

**Figure 3:**
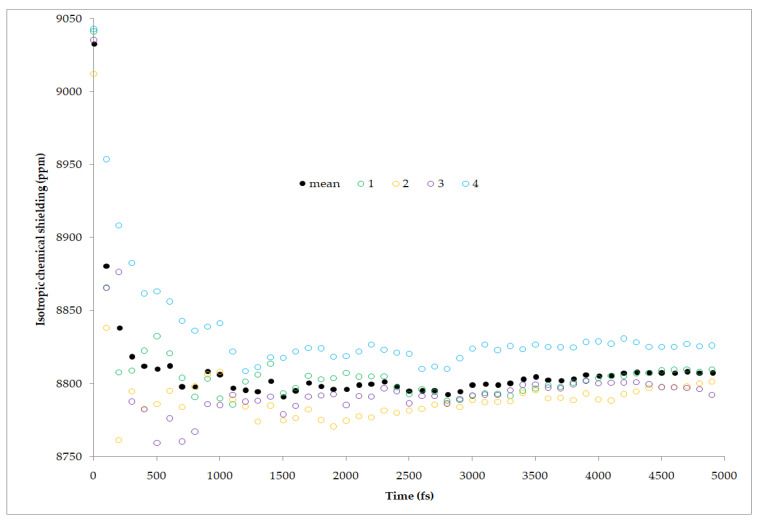
The convergence of the isotropic shielding for the Pb atom in Pb(NO_3_)_2_ with respect to the simulation time. The calculations were performed at 295 K, and the shielding values for the four equivalent atoms in the unit cell (1–4, color circles) were averaged (mean, black disk). Source: author’s personal archive.

Not without a good reason, as the object of the next computational study the lead nitrate (Pb(NO_3_)_2_) was chosen [53]. Accurate temperature measurement in variable-temperature ssNMR (solid state NMR) may prove quite difficult due to the multiple factors, including MAS frequency, rotor dimensions, and air temperature shifts. It is therefore a common practice to use the reference materials that exhibit a continuous shift of resonance frequency as a function of temperature in order to indirectly measure the temperature inside a rotor. The remarkably sensitive temperature dependence, uniform over a range of at least −130 °C to +150 °C, of the ^207^Pb chemical shift in MAS spectra of this salt provides an excellent method for thermometry in solid-state NMR. However, this phenomenon was not fully explained, which encouraged the authors to conduct research in which the quantum MD under periodic conditions was combined with GIPAW NMR calculations. In that study, multiple MD simulations at different temperatures were performed to check if the experimentally observed linear relationship of chemical shift from temperature can be as well reproduced from calculations. The applied method proved to be successful and accurate, especially if taking into consideration the large range of ^207^Pb chemical shifts in various compounds -over 11,000 ppm (Figure 3).

Another computational investigation concentrated on polymorphism, which can be briefly defined as an ability of a compound to exist in different crystal forms. A good example is a study that focused on piracetam, a popular active pharmaceutical ingredient [54]. In the ^13^C ssNMR spectrum of one of the studied forms, the splitting of some peaks suggested that there is more than one molecule in the asymmetric unit (Z′ > 1), although in the experimental crystal structure obtained from SCXRD measurements, only one molecule was present (Z′ = 1, Z = 4). Therefore, quantum MD combined with GIPAW NMR calculations were used to confirm the quality and correctness of the deposited crystal structure. The obtained computational results supported the presence of only one molecule in the asymmetric unit as the standard deviation of the values of the ^13^C isotropic chemical shielding calculated for the four equivalent atoms were found to be negligible. Finally, the splitting was explained as resulting from exceptionally strong residual dipolar couplings between the ^13^C and ^14^N atoms.

#### 3.3.3. Highly Rigid Systems

The practical applicability of the aiMD method used in conjunction with the DFT NMR calculations can be seen in simulations of strongly disordered systems such as polycrystalline silicates. One of the targets of the research performed on such systems is an increase the material’s conductivity. This can be achieved via the usage of different ions that are disordered over a couple of equivalent positions. This is the reason why numerous experiments must be performed. To keep the cost of the experimental part as low as possible, the in silico calculations are undertaken. For example, in case of a lithium-rich phosphidosilicate [30], lithium and silicate ions are characterized by a high mobility and, depending on the temperature, they occupy the system’s voids differently. As the authors of the paper stressed, application of the BOMD was the only possibility to analyze the inner structure of the silicate and therefore to construct new polycrystalline silicates with the desired physical properties.

A similar experiment refers to the mobility of ^7^Li ions in LiTi_2_(PO_4_)_3_and LiZr_2_(PO_4_)_3_ [36]. A stimulus for this work was huge discrepancies between the results of a static NMR calculations and experimental data previously obtained for the above-mentioned systems. Moreover, the importance of a temperature factor was discussed, as the quadrupolar coupling constant associated with Li ion depends strongly on the temperature. For phases inclosing Ti, the constant grows with temperature as the local symmetry of the system decreases. Meanwhile, for the Zr-containing phases, the Li-associated quadrupolar constant decreases, as the thermal vibrations reduce the anisotropy of the interaction. Application of aiMD has not only proven the presence of a short-range Li mobility but also has delivered an explanation for the disagreement observed between the static calculation results and the experimental NMR spectra. Therefore, it has been plainly shown that in order to properly simulate the NMR spectra, thermal vibrations as well as local motions of atoms must be included. Such opportunity is offered only by aiMD.

Another type of highly rigid system is liposomes. In contrast to the silicates, they are rigid as a whole and change the placement of individual chains or chemical groups regarding the changing parameters of the environment, such as pH. One of the examples are liposils: liposomes covered from the outside by a layer of silica [66]. Local dynamical changes occurring between the silica shell and the phospholipids of a liposome can be fully analyzed only when BOMD is applied. Such research is crucial during the construction of effective drug delivery systems. Interestingly, application of the aiMD in such systems requires a special approach. Even if liposils are calculated in parts, these are still relatively big systems—so to acquire the MD snapshots for the NMR calculation, a large time step must be applied. For example, in the work [44], the time step was set at 2.5 ps. It should be noted that, to the best knowledge of the authors, this is the biggest time step found in the research papers using the BOMD and DFT-NMR method (see Table 3). Additionally, from the technical point of view, it should be noted that for such large time steps, the usage of ^3^H in BOMD is a standard procedure. It is implemented in order to avoid too large displacements of the hydrogen atoms, which could happen if both ^1^H and a large time step were applied in one calculation.

## 4. Discussion

aiMD simulations coupled to calculations of NMR parameters have been of great utility in predicting the effects of nuclear vibrations on NMR chemical shieldings and J-couplings in each state of matter. Among the nuclei studied using this combined approach, popular ones such as ^1^H, ^13^C, or ^15^N, but also more exotic, for example, ^131^Xe, ^195^Pt, and ^207^Pb, can be found. It was also confirmed that with aiMD, it is possible to investigate relaxation phenomena as from a single or a set of computed aiMD trajectories, the relaxation times T_1_ and T_2_ can be determined.

In most of the reviewed works, universal and well validated DFT functionals, such as GGA PBE or BLYP and B3LYP, were used. It is believed that if the functional is proven to be accurate in geometry optimization of a specific system, it will be also the proper one to be used in the aiMD simulation. As stated in the introduction, aiMD can be performed at any temperature and pressure. While most of the simulations were performed under standard conditions, some of them were also conducted under increased p or T to adjust to the NMR experimental conditions. Additionally, the simulations for glasses are often performed under increased T in order to better probe the potential energy surface.

In addition, it is worth mentioning that while in the experimental NMR approach in solution a full set of correlation spectra is available, allowing for a deep structural analysis of molecular systems, the use of such techniques for the analysis of solids spectra is currently being developed and is not a routine technique. Therefore, the QM shielding calculation methods are one of the basic approaches used in the analysis of NMR spectra in a solid.

It was confirmed that including fast, small-amplitude motions has a noticeable effect on the accuracy of NMR parameters calculations. In the studies where the authors used both ab initio and molecular mechanics simulations. the general and expected conclusion was always that the ab initio simulations provide more realistic internuclear forces and therefore more accurate time evolution of the dynamic system than any empirical force field. Furthermore, aiMD allows for anharmonic vibrations, whereas most of the current molecular mechanics force fields are based on harmonic approximation.

The common procedure to use the trajectory generated from the aiMD simulations consists of taking the chosen snapshots and using them as the structures for the NMR parameters calculations without the prior geometry optimization (Figure 3). In most of the studied cases, the snapshots were taken systematically, i.e., every 50 fs. In some works, the authors allowed the studied system to adapt to the temperature of simulation through initial equilibration and started collecting snapshots after the specified time. In the other studies, the collecting of snapshots started from the beginning of simulation. Usually, the results are presented in the graphical forms as a running average of the isotropic shielding for the selected atom with respect to the simulation time. The final running average, obtained at the end of simulation, is often referred to as a dynamics-averaged chemical shielding (Figure 3). For disordered systems, aiMD simulations can be also employed to obtain a sample glass structure prior to the GIPAW calculations of NMR parameters.

The computational cost of DFT aiMD allows the simulation of trajectories over several tens of picoseconds (from 1 to 106 ps, Table 1) for systems of a modest size. In all of the reviewed cases, this was found to be sufficient enough for capturing fast vibrational motions and for the calculated NMR parameters to reach their plateaus. The usually applied time step ranges from 0.1 fs up to 1 fs. The choice of the time step depends on type of aiMD (BOMD or CPMD), studied system (presence of H atoms usually implies shorter time steps), and algorithm used for integration in the specific software. Applied types of aiMD with regards to the used software are presented in Table 3. It is not a common practice to present the information on the time needed to obtain the results of calculations, as it obviously depends on the computational power of the available unit. Therefore, it is hard to compare the standard NMR parameters calculations with those preceded by aiMD in this regard. However, from our experience based on the solid state and gas phase modeling, we can estimate that it usually takes about one to two orders of magnitude longer to use combined aiMD and NMR parameters calculations approach than to perform quantum chemical NMR properties calculations on the optimized structure at 0K. This elongation of computations is, in our opinion, the major disadvantage of such a combined approach. However, with the expected increase of the computational power in the future, it is expected that aiMD/QC NMR combinations will develop. The possible expansions of this approach include the application of more demanding aiMD methods (Figure 3), as well as elongation of the simulation time in order to include not only vibrational but also rotational and translational motions to computationally simulate phenomena such as phase transitions or polymorphic transformations.

## 5. Conclusions

In this review, we have shown that the application of aiMD is now not only possible but also in some cases the method of choice to properly calculate the NMR properties for various systems. aiMD, being significantly more accurate than any type of forcefield based dynamics, seems to be the most straightforward method allowing to include fast and small-amplitude vibrational motions for the purpose of subsequent quantum chemical NMR calculations. The possibility to perform aiMD at any pressure and temperature allows to explain at the molecular level the changes in the NMR analysis results originating from the changes of the experimental conditions. From our experience, based on the studies cited in this review, it is justified to say that the computational time needed to involve the aiMD is usually reasonable and calculations can be done on widely available servers.

In order to prepare this review, while carefully studying previously reported works on the application of NMR parameters calculations using quantum chemical methods, we found a few in which the authors reported significant inconsistencies between the theoretical and experimental data. This was sometimes explained as resulting from inability to include the dynamics into the calculations. For example, in [79], the authors state: “It is possible that these motions do not result in locally isotropic symmetry on the time scale of the quadrupolar coupling, but molecular dynamics simulations are required to explore this issue in more detail”. This work was published in 2012, when combination of aiMD and NMR parameters calculations was not yet so accessible. However, we believe that the approach described in this review will become more popular and allow to solve the problems and answer the questions that, until recently, were not possible to address.

## Figures and Tables

**Table 1:** Computational details of literature examples of aiMD combined with DFT-NMR calculations studies. The systems analyzed in a solid state are bolded.

Ref. in Article	Software (MD)	Software (NMR)	Functional (MD)	Functional (NMR)	NMR-Investigated Nuclei and Investigated System	Type of MD	Integration Time Step	Total Simulation Time	Simulation Temperature	Solvation Model
[29]	CASTEP	CASTEP	B3LYP	GGA PBE	^15^N in 5-azopyrimidines	PIMD	0.5 fs	5 ps	300 K	PCM
[30]	CP2K	CASTEP	Goedecker-Teter-Hutter	GGA PBE	Li in polycrystalline Li_14_SiP_6_	BOMD	0.5 fs	5 ps	1023 K	non applicable
[31]	VASP	CASTEP	GGA PBE	GGA PBE	^19^F, ^23^Na, ^45^Sc in melted NaF-ScF_3_ (glass)	BOMD	1 fs	1 ns	900 K–1660 K	non applicable
[32]	CASTEP	CASTEP	GGA PBE	GGA PBE	^1^H, ^13^C, ^14^N, ^17^O, ^35^Cl in amino acids	BOMD	0.5 fs	5 ps	300 K	non applicable
[33]	CASTEP	CASTEP	GGA PBE	GGA PBE	^1^H, ^13^C in l-alanine and beta-l-aspartyl-l-alanine	BOMD	1 fs	3.2 ps	293 K	non applicable
[34]	CPMD	CASTEP	BLYP	no information provided	^1^H, ^15^N, ^13^C in glycine	BOMD	0.29028 fs	96ps	300 K and 427 K	non applicable
[35]	CPMD	Gaussian	B3LYP	B3LYP	^15^N, ^13^C in alanine	BOMD	0.09676 fs	10 ps	300K	PCM
[36]	CASTEP	CASTEP	GGA PBE	GGA PBE	^7^Li in LiR_2_(PO4)_3_ (R= Ti and Zr)	BOMD	1 fs	5 ps	300 K and 4050 K	non applicable
[37]	CASTEP	CASTEP	GGA PBE < B3LYP	GGA PBE	^15^N in guanine-cytozine dimers and isocytosine (monomer, dimer)	CPMD	0.5 fs (monomer, solid state), 0.72 fs (liquid, dimer)	9 ps	300 K	non applicable
[38]	Quantum Espresso	CASTEP	BLYP	GGA PBE	^23^Na in methylamine solution of [Na+ [2.2.2]cryptand Na-] †	CPMD	no information provided	20 ps	258 K	COSMO
[39]	CP2K	ReSpect, ADF	GGA PBE	GGA PBE, KT2	^1^H in [Ir_6_(IMe)_8_(CO)2H_14_]2+	BOMD	0.25 fs	30 ps (complex I), 40 ps (complex II)	298 K	PCM, COSMO
[40]	Quantum Espresso	ADF	GGA PBE	GGA PBE	^127^I, ^131^Xe, ^133^Cs	CPMD	0.145 fs	25 ps for Xe and Cs+, 50 ps for I-	350 K	COSMO and explicit
[41]	VASP	VASP	PBE<<B3LYP	GGA PBE	^15^N	BOMD	0.5 fs	10 ps	298 K	implicit
[42]	Quantum Espresso	ADF	GGA PBE	GGA PBE, PBE0	^7^Li, ^23^Na, ^35^Cl, ^81^Br,^127^I	CPMD	0.121 fs	20 ps	300 K	explicit
[43]	CPMD	CASTEP	BLYP	B3LYP	^1^H, ^13^C, ^14^N, ^17^O in N-methyl acetamide	CPMD	0.09676 fs	106 ps	300 K	PCM and explicit
[44]	VASP	PARATEC	PW	GGA PBE	^1^H, ^31^P in silica-encapsulated liposomes	BOMD	2.5 fs	4 ps	300 K	non applicable
[45]	VASP	Gaussian09	GGA PBE	B3LYP	^7^Li in hydrated Li	BOMD	0.5 fs	45.2 ps	400 K	PCM and explicit
[46]	Quantum Espresso	ADF	GGA PBE	GGA PBE, LDA	^195^Pt in cisplatin	CPMD	5 a.u. (0.122 fs)	5ps NVT + 12ps NVE	300 K	COSMO and explicit
[47]	Turmobole	ADF	GGA PBE	GGA PBE	^199^Hg in [Hg(CN)_2_], [CH_3_HgCl]	BOMD	40 a.u. (0.978 fs)	9 fs (1H), 13 ps (2H)	300 K and 305 K	COSMO and explicit
[48]	CPMD	CPMD	BLYP, GGA PBE	BLYP, GGA PBE	^1^H in water	CPMD	no information provided	10 ps	330 K	non applicable
[49]	CP2K	NWChem	GGA PBE	GGA PBE	^7^Li in Li_2_S_4_, Li_2_S_6_, Li_2_S_8_	BOMD	1 fs	5 ps	298.15 K, NPT (1 atm)	COSMO
[50]	Quantum Espresso	ADF	GGA PBE	GGA PBE	^14^N in neat acetonitrile and 1-methyl-1,3-imidazole and 1-methyl-1,3,4-triazole in different solvents	CPMD	0.145 fs	50 ps for water, 60 ps for benzene, 40 ps for neat acetonitrile, 20 ps for water and benzene systems	350 K	explicit
[51]	Turbomole	Turbomole	B3LYP, RIMP2	B3LYP, RIMP2	^1^H in phosphoric acid	BOMD	1 fs	20,000 fs	298–430 K	COSMO
[52]	FEMTECK	Quantum Espresso	GGA PBE	GGA PBE	^11^B, ^17^O, ^7^Li in molten 0.3Li_2_O–0.7B_2_O_3_ (glass)	BOMD	1.2 fs	no information provided	1250 K	non applicable
[53]	CASTEP	CASTEP	GGA PBE	GGA PBE	^207^Pb in Pb(NO_3_)_2_	BOMD	0.5–1.0 fs	From 5 to 20 ps	various	non applicable
[54]	CASTEP	CASTEP	GGA PBE	GGA PBE	^13^C in piracetam	BOMD	1.0 fs	5 ps	298 K	non applicable
[55]	Quantum Espresso	ADF	PW91 XC	B1PW91	^17^O in UO_2_^2+^, UO_2_(CO_3_)_3_^4−^, (UO_2_)_3_(CO_3_)_6_^6-^	CPMD	0.122 fs	8.5 ps	330 K	COSMO
[56]	CPMD	CPMD	BLYP	BLYP	^13^C in Asp 25-Asp25′ dyad in pepstatin A/HIV-1 protease	CPMD	0.0096 fs	2 ps	no information provided	no information provided

**Table 2:** Influence of the vibrational contribution to the analyzed NMR parameter vs. static DFT-NMR calculations [32].

Analyzed NMR Parameter	Influence of the Vibrational Contribution to the Analyzed Parameter vs. Static DFT-NMR Calculations
Isotropic chemical shift	Increase
Chemical shift anisotropy	Almost no changes (close to the experimental error)
Quadrupolar coupling	Decrease, closer to the experimental data

**Table 3:** Software applied in aiMD combined with the DFT-based NMR calculations. “+” indicates that this is possible to request the NMR parameters calculation in that software.

N°	Software/Code	Type of aiMD	NMR Parameters Calculation	License Type	Ref. Method	Ref. in Article
1	CASTEP	BOMD, PIMD, CPMD	+	Academic, Commercial	[67,68]	[28,31,32,35,36,52,53]
2	CP2K	BOMD	+	Free, General Public License (GPL)	[69,70]	[29,37,48]
3	CPMD	BOMD, CPMD	+	Academic	[71,72]	[33,34,42,47,56]
4	Turbomole	BOMD	+	Commercial	[73,74]	[46,50]
5	Quantum Espresso	CPMD	+	Free, General Public License (GPL)	[75,76]	[37,39,41,45,49,55]
6	VASP	BOMD	+	Academic, Commercial	[77,78]	[30,40,43,44]

## Data Availability

Not applicable.
